# Peering into the ‘black box’ of pathogen recognition by cellular autophagy systems

**DOI:** 10.15698/mic2015.09.225

**Published:** 2015-08-22

**Authors:** Shu-chin Lai, Rodney Devenish

**Affiliations:** 1 Department of Biochemistry and Molecular Biology, Monash University, Clayton campus, Melbourne, Victoria 3800, Australia.

**Keywords:** autophagy, E3 ligase, galectin-8, tripartite motif proteins, ubiquitin

## Abstract

Autophagy is an intracellular process that plays an important role in protecting eukaryotic cells and maintaining intracellular homeostasis. Pathogens, including bacteria and viruses, that enter cells can signal induction of selective autophagy resulting in degradation of the pathogen in the autolysosome. Under such circumstances, the specific recognition and targeting of the invading pathogen becomes a crucial step for the subsequent initiation of selective autophagosome formation. However, the nature of the signal(s) on the pathogen surface and the identity of host molecule(s) that presumably bind the signal molecules remain relatively poorly characterized. In this review we summarise the available evidence regarding the specific recognition of invading pathogens by which they are targeted into host autophagy pathways.

## INTRODUCTION

Macroautophagy (hereafter referred to as autophagy) is an intracellular process that plays an important role in protecting eukaryotic cells and maintaining intracellular homeostasis. Canonical non-selective autophagy generally functions to help the cell overcome stress conditions, such as nutrient starvation, by forming double-membrane autophagosomes containing cellular components which subsequently fuse with lysosomes (in mammals), such that the contents within are degraded and released back to the cytosol in order to maintain nutrient levels 1. However, autophagy can be selective whereby specific cellular components are recognized, such as damaged mitochondria (mitophagy), in response to specific stimulatory signals 2. Apart from internal metabolic signals, recent research has found that pathogens, including bacteria and viruses, that enter cells can signal induction of autophagy resulting in degradation of the pathogen in the autolysosome, or triggering downstream innate immune responses, as well as the presentation of antigen to start the adaptive immune responses 3. Under such circumstances, the selective recognition and targeting of the pathogen becomes a crucial step for the subsequent initiation of selective autophagosome formation. However, the nature of the signal(s) on the pathogen surface and the host molecule(s) that presumably bind the signal molecules remain relatively poorly characterized. Here we summarise the available evidence regarding the specific recognition of invading pathogens by which they are targeted into host autophagy pathways in mammalian cells.

## UBIQUITINATION ACTS AS AN ‘EAT-ME’ SIGNAL THAT TARGETS BACTERIAL PATHOGENS

Ubiquitination is a post-translational modification of proteins that is involved in several crucial cellular functions 4. Recent reviews have comprehensively covered what is understood about the mechanism of ubiquitin-dependent selective autophagy recognition, by which the intracellular cargo is ubiquitinated and subsequently interacts with the autophagosomal membrane component LC3 2,5. These interactions are mediated by autophagic receptors referred to as sequestosome-like receptors (SLRs; 3) and include the eponymous sequestosome 1 (SQSTM1/p62, hereafter referred as p62), neighbour of BRCA1 gene 1 (NBR1; 6), nuclear domain 10 protein 52 (NDP52; 7), NDP52-like receptor calcoco3 (also known as Tax1bp1; 8), and optineurin (OPTN; 9). These SLRs are characterised by the existence of both an ubiquitin-binding domain (UBD) and a LC3-interacting region (LIR) in their structure which endow the ability to interact with both ubiquitin and LC3 5. There is also a considerable body of literature concerning the mechanisms by which pathogens circumvent autophagy recognition (see reviews by Ashida *et al.* 10, and Huang and Brumell 11) which will not be considered further here. We now address the available information regarding those pathogen-associated targets known to be ubiquitinated.

The ubiquitination of a protein target is mediated by members of the E3 ubiquitin ligase superfamily comprised of four major families: Really Interesting New Gene (RING), homologous to E6-associated protein C-terminus (HECT), UFD2 homology (U-box), and RING-in-between-RING (RBR). Each class of E3 ligase facilitates transfer of the ubiquitin moiety to the protein target by a different mechanism (reviewed by Davis and Gack 12). Apart from the conserved domain that is responsible for ubiquitin conjugation, E3 ligases also contain multiple additional domains that are related to the recruitment of the target substrate 13. Therefore, in order to attach ubiquitin to an invading pathogen, presumably the E3 ligase must have a specific domain capable of recognising at least one surface component of the pathogen, or alternatively one or more host components that are specifically associated with the pathogen. However, despite early observations that indicated bacteria 14, or damaged membranes associated with bacteria could be ubiquitinated, subsequent intense investigations have only revealed the identity of such E3 ligases in one or two cases.

LRSAM1 is a RING E3 ligase that contains a N-terminal leucine-rich receptor (LRR) domain and C-terminal RING domain by which ubiquitin is ‘added’ to intracellular *Salmonella enterica* serovar Typhimurium (hereafter *Salmonella*) that have escaped from phagosomes leading to their elimination by autophagy 15. The LRR domain is present in several pathogen recognition receptors (PRRs), such as toll-like receptors (TLRs) and NOD-like receptors (NLRs), facilitating recognition of a variety of pathogen associated molecular patterns (PAMPs) and initiating downstream immune responses 16. The LRR and RING domains in LRSAM1 were reported to be essential for detecting bacteria and assembling ubiquitin, respectively 15,17. Presumably, LRSAM1 utilises its LRR region to recognise the conserved regions of surface components of bacterial pathogens. However, their identity remains unknown. Likewise the identity of any bacterial proteins ubiquitinated by LRSAM1 also remains to be established. An attempt to identify bacterial proteins ubiquitinated by application of a mass spectroscopy approach was not successful 15. However, evidence of auto-ubiquitination of LRSAM1 at internal lysine residues was found suggesting that auto-ubiquitination leads to subsequent p62 recruitment 15. The stimulus for auto-ubiquitination is currently unknown. While ‘early’ events in bacterial recognition remain unclear, with a role for NDP52 in LRSAM1 recruitment being excluded 15, it is known that ubiquitin, NDP52 and p62 are subsequently recruited to bacteria and damaged vacuoles (see below).

Parkin is the second ubiquitin E3 (RING-in-between-RING) ligase for which there is evidence that it can be recruited to intracellular bacteria 18. Notably, Parkin was first described as having a role in the selective clearance of dysfunctional mitochondria (by mitophagy 19,20). *Mycobacterium tuberculosis* can survive within macrophages by blocking phagosome maturation 21. The mycobacterial early secretory antigenic target 6 system 1 (ESX-1) permeabilises the phagosome membrane and exposes the bacteria within to the cytosol. The ubiquitination detected occurred while bacteria remained resident within phagosomes. Therefore, it seems Parkin catalyses Lys63-linked polyubiquitination of *M. tuberculosis*-containing phagosome membranes leading to the recruitment of the autophagy receptors p62, NBR1 and NDP52, and the subsequent delivery of the ‘marked’ membranes to autophagosomes 18. This scenario indicates that the substrate ubiquitinated by Parkin must be host cell-intrinsic, most likely one or more protein components of the bacteria-containing phagosome membrane. Again the identities of the actual protein targets remain to be determined. There is no evidence for the involvement of LRSAM1 in ubiquitination of *M. tuberculosis*.

## ‘DOUBLING-UP’ WITH FAT10?

Recently the ubiquitin-like protein FAT10 has been shown to be recruited to cytosolic *Salmonella* following their release from vacuoles in infected human cells 22. FAT10 is composed of two ubiquitin-like domains and possesses a free C-terminal di-glycine motif that is required for the formation of FAT10 conjugates. The FAT10-decorated bacteria were simultaneously decorated with ubiquitin, p62, NDP52 and LC3B. While FAT10 co-localized with p62-positive microdomains on *Salmonella*, the co-localization with NDP52 was only partial suggesting that FAT10 is not involved in sensing damaged membranes of vacuoles (from which the bacteria have escaped) through galectin-8 binding which can recruit NDP52 23, see below. Furthermore, co-localization with ubiquitin was “intermediate” between that found for p62 and NDP52 22. However, it remains unclear whether FAT10 binds directly to bacterial structures, or to cellular structures proximal to, or surrounding them.

The authors of this study 22 raise the question as to why cells might ‘double up’ and utilise an anti-bacterial strategy involving two ubiquitin family proteins that both bind to p62. They note earlier findings that FAT10 is only expressed in IFN-γ or TNF-α stimulated cells and in mature dendritic cells. Interestingly, they suggest the reason may relate to the fact that ubiquitin needs to assemble into chains on a target, whereas FAT10 with its two ubiquitin-like domains can serve as a degradation signal without the need for chain formation 24. In the case of ubiquitin chains these can be interfered with by pathogen-encoded deubiquitinases, on the other hand FAT10-deconjugating enzymes have not been described and bacteria cannot interfere with FAT10 action by suppressing chain formation. A corollary of this may be that FAT10 and ubiquitin modify different proteins on the surface of *Salmonella*.

## DAMAGED VACUOLES EXPOSE CELLULAR GLYCANS THAT ARE TARGETED BY GALECTIN-8

Pathogen-containing phagosome membranes contain a variety of host-intrinsic components including surface receptors, glycans, phospholipids and cholesterols, many of which act to initiate downstream signal transduction as a part of host response to the infection. Galectin-8 (Gal-8) is a member of the β-galactoside-binding protein family (galectins) which can bind glycans to regulate several immune cell processes including pathogen recognition and cellular adaptive immune and inflammatory responses 25. Following infection of epithelial cells (HeLa, CHO and HEK293T) by *Salmonella *a subset of intracellular bacteria are marked by Gal-8 binding followed by NDP52 binding and LC3 recognition. The bacteria-containing phagosomes must have suffered at least a partial loss of membrane integrity leading to exposure of the β-galactoside located in the inner leaflet of the membrane which is then targeted by Gal-8. The targeted glycan signal was verified as being host derived rather than microbial because the binding assay showed that Gal-8 failed to interact with free *Salmonella*. The direct binding between Gal-8 from bacteria-associated phagosome fragments and NDP52 leads to recognition for autophagosome formation 23. More recently, this model has been proposed to require the activity of a family of guanylate-binding proteins (GBPs) belonging to the interferon-inducible GTPase superfamily and which are recruited to, and necessary for, the lysis of pathogen-containing vacuoles 26. Interestingly, there is also other evidence that indicates individual members of this GBP family interact with autophagy effectors (GBP1 interacts with p62; GBP7 binds Atg4B), suggesting they make additional contributions to the cellular defence against intracellular bacteria 27.

## Atg5 AND Tecpr1 ARE INVOLVED IN AUTOPHAGY TARGETING of *Shigella flexneri*

Pathogens might enter the autophagic pathway by interacting with autophagy proteins other than LC3. Tectonin domain-containing protein (Tecpr1) selectively targets invading pathogens for autophagy, in addition to damaged mitochondria and protein aggregates, by direct binding to Atg5 28. The Gram-negative bacterium, *Shigella flexneri*, can be targeted by autophagy through the direct interaction between Atg5 and bacterial surface protein, IcsA 29. Normally the secreted bacterial effector protein, IcsB, competitively binds to IcsA thus allowing bacteria to avoid recognition by Atg5. In bacteria lacking IcsB, Atg5 can bind IcsA and target the bacteria for autophagy^30^. This process seems to require Tecpr1 in order to bridge between Atg5-targeted bacteria and the WIPI2-positive phagophore membrane 31. It would seem that in this scenario Atg5 itself acts as a sensor to detect and bind bacteria followed by the recruitment of Tecpr1 and WIPI2. Tecpr1 also binds to the Atg5-Atg12 complex later in phagophore elongation and ultimately contributes to autophagosome maturation. To date, this model is best described in terms of the recognition of *S. flexneri*. However, Ogawa *et al.* 28 have reported that Tecpr1 is involved in the targeting of *Salmonella* and Group A *Streptococcus* to autophagosomes.

## SMAD SPECIFIC E3 UBIQUITIN LIGASE 1 (SMURF-1) AND DELIVERY OF VIRAL CAPSID PROTEINS TO AUTOPHAGOSOMES

Autophagy also plays a role in antiviral immunity 32,33,34. There is evidence that autophagy functions in a protective role in cells infected by the lethal central nervous system Sindbis virus (SV). The receptor p62 directly interacts with SV capsid protein, but not the envelope glycoprotein, and is required for targeting SV to autophagosomes 35.

Recently, the HECT family SMAD specific E3 ubiquitin ligase 1 (SMURF-1) has been associated with selective autophagy targeting both viral capsid proteins and mitochondria 36. (The role of SMURF-1 in initiating mitophagy will not be considered here). SMURF-1 interacts directly with the SV capsid protein and this association is required for the subsequent recruitment of LC3. There is no evidence for the transfer of ubiquitin in the recognition process. The recognition of capsid by SMURF-1 may be mediated by an N-terminal membrane-targeting C2-domain, although this remains to be formally demonstrated. The C-terminal HECT-domain of SMURF-1 is essential for its function as an E3 ligase 37.

Furthermore, Orvedahl *et al.* 36 have demonstrated that cells lacking expression of SMURF-1 lose the ability to degrade mutated herpes simplex virus type I (HSV-1) harbouring the virophagy inhibitor gene (ICP34.5) deletion, suggesting SMURF-1 may also target this double-strand DNA virus (herpes virus) in a manner similar to the positive-strand RNA virus, SV. By contrast, following infection by Chikungunya virus (CHIKV) p62 binds ubiquitinated capsid and targets it to autophagolysosomes, but the ubiquitination appears not to be dependent on SMURF-1 38.

## TRIPARTITE MOTIF PROTEINS (TRIMs), AN E3 LIGASE FAMILY WITH AUTOPHAGY-RELATED FUNCTIONS

The TRIM family contains over 70 members in human cells which are recognised by the presence of an N-terminal consensus RBCC structure (that consists of a RING, one or two B Boxes and a Coil-Coil domain), while the C-terminal domain varies between each subgroup 39. It has been demonstrated that particular members of the TRIM family play a role in the host response to infection by several viral pathogens 40. As autophagy is now recognised as a mechanism by which virus can be cleared from inside infected cells, it is perhaps not surprising that TRIM proteins have recently been implicated in host autophagy response to viral infection as representatives of a new class of autophagy receptors 41.

TRIM5α provides the best characterised example to date of autophagy elimination of a viral pathogen mediated by direct recognition of a viral capsid protein. TRIM5α, contains the common N-terminal RBCC motif and a C-terminal protein-protein interaction PRY-SPRY domain 39. The PRY-SPRY domain of TRIM5α was previously shown to be required to restrict human immunodeficiency virus-1 (HIV-1) in rhesus monkeys 42. Furthermore, TRIM5α was known to directly interact with recombinant HIV-1 viral capsid to promote the rapid uncoating of virus particle, which is detrimental to the infection and restricts virus dissemination upon infection of HeLa cells 43. The new finding is that TRIM5α serves as an autophagic receptor and directly recognizes (i.e., without a need for ubiquitin tags) the retroviral p24 capsid and delivers it to autophagosomes for degradation 41.

TRIM5α binding to capsid mediates interactions with several autophagy-associated components, including p62, ULK1 and Beclin 1 41. Interestingly, TRIM5α also binds to LC3 family members, preferentially GABARAP, but is unable to bind LC3B the LC3 isoform that most commonly participates in autophagosome formation 41. It is likely that reports of further examples of TRIM family members acting as autophagy receptors in viral infections will be forthcoming, because as indicated above several other TRIM family members have been shown to be critical factors that restrict virus replication and initiate a series of anti-viral immune responses 39. Moreover, over half of the human cohort of TRIMs is reported to regulate autophagy 41.

To date the only example of a TRIM family member implicated in the recognition of intracellular bacteria is TRIM21, a close homolog of TRIM5α. TRIM21 is a type of cytosolic Fc receptor able to recognise intracellular antibody directed against a pathogen or the antibody-bound pathogen itself that has entered the cell via phagocytosis. McEwan *et al.* 44 found that a subset of antibody-bound *Salmonella* was co-localised with TRIM21 four hours after infection of HeLa cells, indicating TRIM21 may also target bacterial pathogens. The crystal structure of TRIM21 revealed a canonical binding interface within its PRY-SPRY domain for the Fc region of immunoglobin G 45 and presumably it is the interaction of TRIM21 with the antibody component of antibody-bound *Salmonella *that mediates its clearance via autophagy. The RING domain of TRIM21 that is responsible for its E3 ubiquitin ligase function, catalyses formation of free K63 ubiquitin chains required for downstream NFκB-stimulated inflammatory response. Notably, Lys63-linked polyubiquitination (see above) has been reported to be associated with ubiquitin-dependent selective autophagy 5,18. It should also be noted that TRIM21 is rapidly recruited to antibody-bound non-enveloped virus and targets it to proteasomes via its E3 ubiquitin ligase activity 46.

## A TRIM56 AND STING CONNECTION?

During infection of macrophages by *M. turberculosis* while intracellular bacteria are generally regarded to reside in intact phagosomes, some gain access to the cytosol (see above) and elicit the host cytosolic DNA sensing pathway by bacteria-intrinsic extracellular DNA (eDNA) 47. The source of eDNA is apparently the surface of bacterial cells in agreement with reports that eDNA is required for the formation of biofilms and outer membrane vesicles during bacterial growth 48,49. *M. tuberculosis* eDNA exposed to the cytosol is recognised by stimulator of interferon gene 1 (STING), leading to the recruitment of ubiquitin followed by initiation of autophagy 50. Recognition of *M. tuberculosis* eDNA also involves TANK-binding kinase 1 (TBK1) 51. Interestingly, STING has been shown to be targeted by TRIM56 which initiates Lys-63 ubiquitination that is required both for the activation of TBK1 and subsequent type I IFN gene transcription 52. A potential connection then, is that TRIM56 catalyses the ubiquitination of STING-targeted *M. tuberculosis* via the exposed eDNA which subsequently recruits p62, NDP52 and TBK1 to mediate autophagy of the targeted bacteria.

## SLR-INDEPENDENT AUTOPHAGY INDUCTION

In *Salmonella* infection the lipid, diacylglycerol (DAG), can co-localize to bacteria-containing compartments and mediate targeting to autophagosomes independently of ubiquitination, or p62 co-localization 53. The detailed mechanism of this mode of autophagy induction remains to be uncovered, although it is known to be dependent upon *Salmonella* pathogenicity island-1 type three secretion systems suggesting that membrane damage of the bacteria-containing compartment by secreted effectors is a key step in autophagy induction.

A single report 54 has shown that 8-nitroguanosine 3',5'-cyclic monophosphate (8-nitro-cGMP) can mark intracellular group A Streptococcus (GAS) via S-guanylation. Such bacteria were also marked with Lys63-linked polyubiquitination leading to subsequent capture of GAS in autophagosomes. There is no information currently available as to whether other intracellular bacteria are targeted to autophagy by S-guanylation.

Atg16L has been demonstrated to interact with NOD1/2, a type of PAMP (pathogen associated molecular pattern), which recruits the Atg12-Atg5-Atg16L complex to the bacteria entry site 55. Also, it is evident that Atg16L can target ubiquitinated Salmonella-containing phagosomes by means of direct interaction between its C-terminal WD β-propeller domain that directly binds ubiquitin and deliver these phagosomes to autophagy in a LC3-independent manner 56.

## CONCLUSION

**Figure 1:**
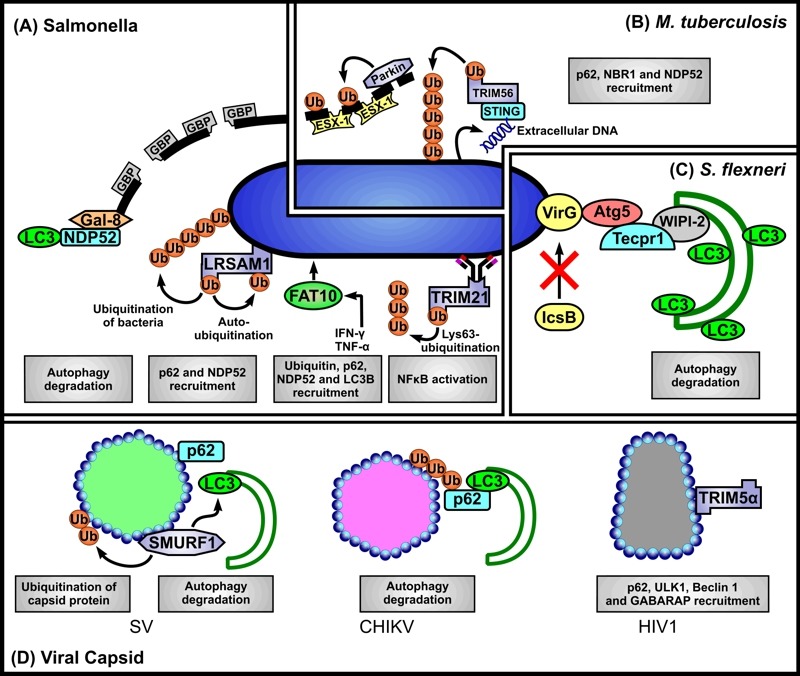
FIGURE 1: Scenarios for SLR-dependent autophagy induction. ** (A)** *Salmonella*. Autophagic clearance of *Salmonella *that have ‘escaped’ from phagosomes, or reside in permeabilised phagosomes may be mediated via several scenarios. First, *Salmonella*-containing phagosome membrane segments that occur as a consequence of loss of membrane integrity, in part mediated by guanylate-binding proteins (GBPs), are targeted by direct binding of galectin 8 (Gal-8) and subsequent binding of NDP52 leading to their recognition as autophagosome cargo. Second, the E3 ligase LRSAM1 can recognise as yet unidentified conserved regions of bacterial surface components. Bacterial proteins are ubiquitinated by LRSAM1, but their identity also remains to be established. Auto-ubiquitination of LRSAM1 at internal lysine residues is implicated in p62 recruitment. Additionally NDP52 is recruited to bacteria. Third, simultaneous decoration of bacteria with FAT10, ubiquitin, p62, NDP52 and LC3B may occur, but it is unclear whether FAT10 binds directly to bacterial structures, or to cellular structures proximal to, or surrounding them. Fourth, antibody-bound bacteria may interact with TRIM21 whose RING domain catalyses formation of free K63 ubiquitin chains required for downstream NFκB-stimulated inflammatory response and LC3 recruitment. ** (B)** *M. tuberculosis*. The E3 ligase Parkin catalyses Lys63-linked polyubiquitination of bacteria-containing phagosome membrane segments which have been permeabilised by the secreted mycobacterial effector system ESX-1 (early secretory antigenic target 6 system 1). Subsequently p62, NBR1 and NDP52 are recruited for delivery of the ‘marked’ membranes to autophagosomes. Additionally, bacteria ‘exposed’ to the cytosol can elicit the host cytosolic DNA sensing pathway through bacteria-intrinsic surface-located extracellular DNA that is recognised by stimulator of interferon gene 1 (STING). STING is targeted by TRIM56 which catalyses Lys-63 ubiquitination of STING-targeted bacteria subsequently leading to the recruitment of p62, NDP52 and TBK1 to mediate autophagy. ** (C)** *S. flexneri*. Atg5 binds the bacterial surface protein IcsA of bacteria lacking IcsB (IcsB-) thereby targeting them for autophagy. Tectonin domain-containing protein (Tecpr1) bridges between the Atg5-targeted bacteria and the WIPI2-positive phagophore membrane. ** (D)** Viral capsids. Direct interaction of p62 and of the SMAD specific E3 ubiquitin ligase 1 (SMURF-1) with Sindbis virus (SV) capsid protein is required for the subsequent recruitment of LC3. Chikungunya virus (CHIKV) capsid is ubiquitinated, bound by p62 and targeted to autophagy, however capsid ubiquitination appears to be independent of SMURF-1. The human immunodeficiency virus (HIV) p24 capsid is directly recognised by TRIM5α (without a need for ubiquitination) and delivered it to autophagosomes. TRIM5α binding to capsid also mediates interactions with several autophagy-associated components, including p62, ULK1, Beclin 1 and GABARAP.

Clearly the mechanisms of autophagy recognition of intracellular pathogens are far from fully being understood. The ubiquitin-autophagy receptor-autophagosome protein model seems applicable to several pathogens (see Figure 1), but the target of the initiating ubiquitination events in all cases remains obscure, and the identity of the specific E3 ligase involved has been determined only in two cases (as being LRSAM1 or Parkin). In some instances different autophagy receptors are involved in recognizing intracellular bacterial pathogens, for example *Salmonella* is recognized by p62 and NDP52. Moreover, DAG can also target bacteria-containing compartments. Collectively such observations emphasize the observation made by Deretic *et al.* 33 that autophagic defences are “multilayered and co-operative in nature”. The challenge for the future is to fully document the multiple pathways and those host- or pathogen-related structures that are recognized and lead to targeting of individual pathogens for selective autophagy.
